# New Applications of JAK/STAT Inhibitors in Pediatrics: Current Use of Ruxolitinib

**DOI:** 10.3390/ph15030374

**Published:** 2022-03-19

**Authors:** Annalisa Marcuzzi, Erika Rimondi, Elisabetta Melloni, Arianna Gonelli, Antonio Giacomo Grasso, Egidio Barbi, Natalia Maximova

**Affiliations:** 1 Department of Translational Medicine, University of Ferrara, 44121 Ferrara, Italy; annalisa.marcuzzi@unife.it; 2 Department of Translational Medicine and LTTA Centre, University of Ferrara, 44121 Ferrara, Italy; erika.rimondi@unife.it; 3 Department of Environmental and Prevention Sciences, University of Ferrara, 44121 Ferrara, Italy; arianna.gonelli@unife.it; 4 Department of Pediatric Hematology and Oncology, IRCCS Policlinico Sant’Orsola Malpighi, 40138 Bologna, Italy; agrasso.ageop@aosp.bo.it; 5 Department of Medicine, Surgery and Health Sciences, University of Trieste, 34127 Trieste, Italy; egidio.barbi@burlo.trieste.it; 6 Department of Pediatrics, Bone Marrow Transplant Unit, Institute for Maternal and Child Health-IRCCS Burlo Garofolo, 34137 Trieste, Italy; natalia.maximova@burlo.trieste.it

**Keywords:** Janus kinase, GVHD, ruxolitinib, pediatrics, cytokines, inflammation

## Abstract

Janus kinases (JAK) are a family of tyrosine kinases (JAK1, JAK2, JAK3, and TYK2) that transduce cytokine-mediated signals through the JAK–STAT metabolic pathway. These kinases act by regulating the transcription of specific genes capable of inducing biological responses in several immune cell subsets. Inhibition of Janus kinases interferes with the JAK–STAT signaling pathway. Besides being used in the treatment of cancer and inflammatory diseases, in recent years, they have also been used to treat inflammatory conditions, such as graft-versus-host disease (GVHD) and cytokine release syndrome as complications of allogeneic hematopoietic stem cell transplantation and cell therapy. Recently, the FDA approved the use of ruxolitinib, a JAK1/2 inhibitor, in the treatment of acute steroid-refractory GVHD (SR-aGVHD), highlighting the role of JAK inhibition in this immune deregulation. Ruxolitinib was initially used to treat myelofibrosis and true polycythemia in a high-dose treatment and caused hematological toxicity. Since a lower dosage often could not be effective, the use of ruxolitinib was suspended. Subsequently, ruxolitinib was evaluated in adult patients with SR-aGVHD and was found to achieve a rapid and effective response. In addition, its early low-dose use in pediatric patients affected by GVHD has proved effective, safe, and reasonably preventive. The review aims to describe the potential properties of ruxolitinib to identify new therapeutic strategies.

## 1. Introduction

**Figure 1:**
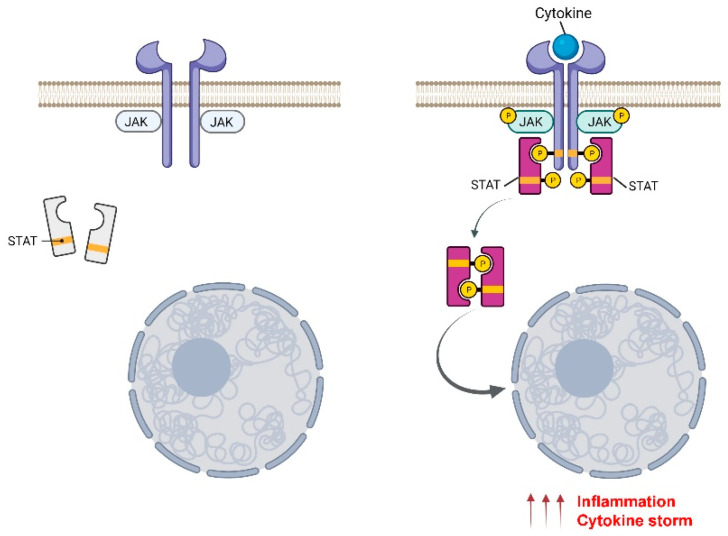
Schematic representation of JAK/STAT signal transduction pathway by cytokines. A cytokine binding to the receptor induces JAK activation and, consequently, STAT phosphorylation. Activated STATs form hetero- or homodimers that translocate to the nucleus and regulate the transcription of genes that cause hyperinflammation and cytokine storm. Created with BioRender.com (accessed on 16 March 2022).

Janus kinases (JAKs) are enzymes that belong to the tyrosine kinase family and play a role in transmitting cell activation signals, induced by growth factors, hormones, or inflammatory cytokines, to transcription factors called STATs (signal transducers of activated transcription), which migrate from the cytosol into the cell nucleus [1] (Figure 1).

The name of these enzymes was given in reference to the two-faced Roman god Janus, for its characteristic to have in the tertiary structure two protein kinase domains, although initially the crucial role of these enzymes was not realized and they were referred to simply as “just another kinase” to distinguish them from the considerable number of identified kinases [2,3,4].

This class of enzymes consists of four members (JAK1, JAK2, JAK3, and TYK2) which have a generic uniform tissue distribution, except for JAK3, which is expressed exclusively at the hematopoietic level. The structure of all JAKs consists of four structural domains composed of seven homologous regions, named JH1-7 (Janus Homology 1–7). JH1 and JH2 correspond, respectively, to the tyrosine kinase and pseudo-kinase domains [5,6].

JAK family members transduce signals interacting with cytokine receptors—integral membrane proteins consisting of an extracellular domain, a transmembrane portion, and an intracellular domain that lacks intrinsic enzymatic capacity. The intracytoplasmic part of these receptors is in contact with JAKs and, through them, performs the catalytic function [7,8]. The receptors are associated with different combinations of JAKs depending on the cytokine. When there is no stimulus, the receptors bind the inactive form of JAK, which undergoes a significant structural change at the intracytoplasmic level in the presence of a ligand (Figure 1). This change activates the JAK molecule and induces binding of the STAT protein, which is activated by a process of auto-phosphorylation, referred to as cross-phosphorylation, in correspondence with their “activation loops”. As a result of this phosphorylation, activated STAT proteins form a dimer that reaches the nucleus and directly regulates the expression of genes related to different biological responses depending on the cell or tissue environment [9,10].

Among the JAK family’s kinases, the most studied are JAK1 and JAK2, first of all because of the important role that they play in the inflammatory response. JAK3, indeed, can exert a more limited role because it exclusively associates with the common receptor chain γ (γc) cytokine family [11,12], which includes interleukin-2 (IL-2), IL-4, IL-7, IL-9, IL-15, and IL-21 [13,14]. JAK1 and JAK2, instead, transduce signals induced also by other cytokines, as interferons, IL-6 or GM-CSF,, which are deeply implicated in the pathogenesis of several immune-mediated diseases [11]. Moreover, while JAK1–3 are associated with inflammatory diseases, JAK1 and 2 signaling, are more strictly connected to malignancies, including myeloproliferative neoplasms [15,16].

In the last few years, several JAK inhibitors have been developed to ameliorate the inflammatory state of different immune-mediated diseases and as cancer therapies. The majority of these compounds, already approved or under the late clinical trial phase, block tyrosine kinase domains, located in the JH1 region of the structure, competing with ATP at the active sites [5]. Kinase domains are involved in inducing phosphorylation and subsequent signal transductions in cells [11]. Other inhibitors are conceived to target the JH2 pseudo-kinase domain, an ideal allosteric site for designing JAK inhibitors with high selectivity [17,18]. Among these drugs, some are classified as non-selective inhibitors, e.g., tofacitinib, which acts preferentially on JAK1 and 2, first approved for the treatment of rheumatoid arthritis (RA) and then for psoriasis and ulcerative colitis, and bariticinib, which also acts on JAK1 and 2 and has been approved for treating RA [11]. Both of these compounds are first-generation ATP-competitive JAK inhibitors; indeed, they act by blocking the ATP-binding pocket in the JH1 tyrosine kinase domain [19,20]. The amino acid sequence within the ATP-binding sites is highly conserved among all JAK kinases and, consequently, the first-generation inhibitors were not strictly selective, targeting several JAK proteins [21]. To prevent the onset of side effects due to the non-specificity of these drugs, the second generation of JAK inhibitors was conceived. These inhibitors, indeed, are defined as selective for their ability to interfere with structurally different regions in the active sites of specific JAK kinases. To this group belong, for example, upadacitinib, the first selective JAK inhibitor approved for RA, and filgotinib, both able to act selectively against JAK1, and decernotinib and ritlecitinib, selective for JAK3. Among the TYK2 targeting drugs, deucravatinicib allosterically and selectively binds to the JH2 domain of this kinase [22]. 

All of these selective drugs are in the late stages of clinical studies for immune-mediated diseases, such as RA, ankylosing spondylitis, Crohn’s disease, psoriasis, etc. [11].

Janus kinases are also of great clinical interest given the possibility of employing them to combat and/or ameliorate acute or chronic graft-versus-host disease (GVHD) [23,24]. Indeed, drugs as itacitinib, a highly selective JAK1 inhibitor, initially developed for the treatment of different malignancies, has been and is currently being tested in different clinical trials for GVHD, as well as bariticinib, which blocks JAK1 and 2—another very promising JAK inhibitor, considering the data obtained in a preclinical murine model [25]. Several completed and ongoing clinical trials for GVHD focused on another encouraging JAK inhibitor, ruxolitinib.

## 2. Ruxolitinib, a Very Promising JAK1/2 Inhibitor

**Figure 2:**
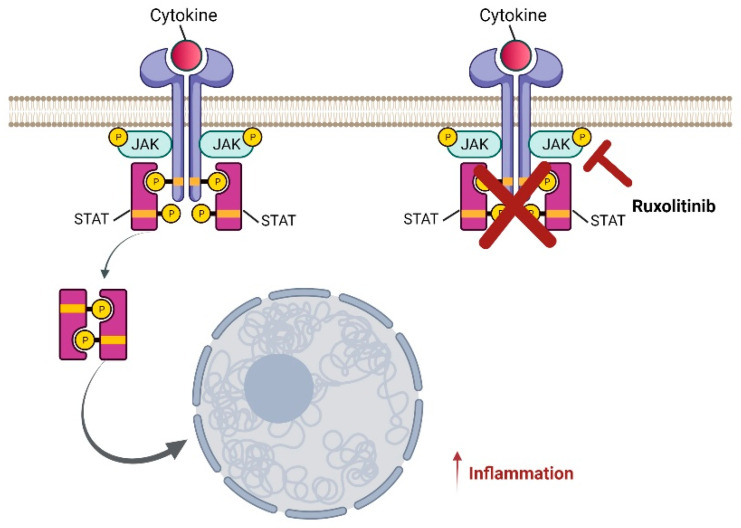
Schematic representation of ruxolitinib’s effect on inflammation induced by the JAK–STAT signal transduction pathway. Ruxolitinib, administered during inflammation, blocks the receptors and triggers the reduction of STAT phosphorylation and, consequently, decreases hyperinflammation and cytokine storm. Created with BioRender.com (accessed on 16 March 2022).

Ruxolitinib is a selective inhibitor of JAK proteins (Figure 2), in particular of JAK1 and 2, and it is a drug that has been indicated for the pharmacological treatment of myelofibrosis and polycythemia, which belong to the group of myeloproliferative neoplasms, whose pathogenesis is associated with the deregulation of the signals associated with these two enzymes [26]. Chronic myeloproliferative diseases, such as true polycythemia, essential thrombocythemia, and primary myelofibrosis, are characterized by mutations affecting JAK family tyrosine kinases. Mutations against JAK1/2 lead, in general, to the dysregulation of the regulatory mechanisms, causing particularly prolonged activity and resulting in the proliferative stimulation of the cell clones involved. This mechanism is more evident when associated with the main function of JAK2 to regulate normal erythropoiesis, both in quantitative and qualitative terms, and mutations at this level lead to abnormal hyperproliferation [27,28,29,30]. 

Clinically, myelofibrosis is characterized by an abnormal proliferation of the megakaryocytic and myeloid lines, with reactive medullary fibrosis and extra-medullary hematopoiesis. Patients with myelofibrosis, indeed, have a rather serious inflammatory symptomatology that is related to cytokine production, and, in many patients, the spleen is enlarged, sometimes considerably, and occupies a significant part of the abdomen. The consequence is a compression of the surrounding organs that causes inappetence and post-prandial clutter sensation. These aspects have a considerable impact on quality of life. Ruxolitinib is an excellent alternative to allogeneic hematopoietic stem cell transplantation, which is, however, the only therapeutic solution for the disease [31]. Treatment of myelofibrosis with ruxolitinib is an example of precision medicine, since this drug is a selective inhibitor of JAK1 and JAK2. Inhibition of JAK1 acts to reduce systemic inflammatory symptoms caused by cytokine storm, while inhibition of JAK2 has among its effects the reduction of neoplastic clones and spleen size.

Treatment with ruxolitinib has recognized effectiveness, but adverse and side effects have manifested significantly. The most common side effects include thrombocytopenia, anemia, neutropenia, urinary tract infections, bleeding, bruising, weight gain, hypercholesterolemia, and increased liver enzyme levels [32,33,34,35]. 

Based on the inhibitory mechanism towards JAK1/2, the Food and Drug Administration (FDA), in 2019, extended approval of the use of ruxolitinib for the treatment of GVHD in acute, steroid-resistant cases in adults and children aged 12 and over.

The need to identify an effective treatment for patients with acute, steroid-resistant GVHD (a complication of hematopoietic bone marrow transplantation) stems from the evidence that they can progress towards a severe disease, with a mortality rate of 70% at one year.

Later, in 2021, the FDA extended the use of ruxolitinib further in adults and children over 12 years of age with chronic GVHD already treated with 1–2 lines of systemic therapy.

## 3. Ruxolitinib Use in Pediatric Patients: Indications and Dosages

**Figure 3:**
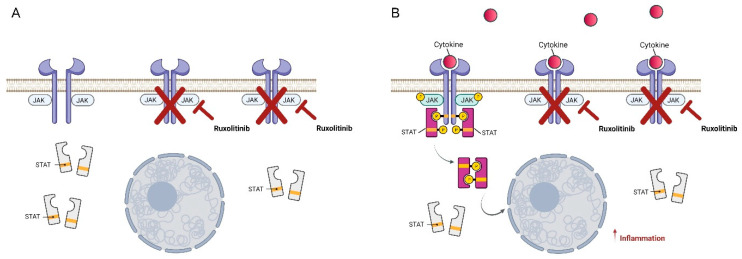
Schematic representation of the preventive anti-inflammatory action of ruxolitinib. (**A**) Ruxolitinib, administered at low dosage and in the absence of cytokine induction, can partially block JAK receptors. (**B**) Partial inhibition of JAK receptors by ruxolitinib reduces STAT activation and prevents hyperinflammation. Created with BioRender.com (accessed on 16 March 2022).

The use of JAK inhibitors, including ruxolitinib, in pediatric settings remained canonically limited to rare therapeutic indications that consisted, essentially, in its anecdotal use in interferonopathies, such as a deficit of DNAse2 or SAVI syndrome, or for relapsed or refractory hemophagocytic lymphocytosis (HLH). In this setting, ruxolitinib was used as a long-life therapy or as bridging to hematopoietic stem cell transplantation (HSCT) with good response (73.5% OR) and acceptable toxicity [36,37]. In the last few years, there has been growing interest in ruxolitinib in the setting of HSCT. In particular, results from the REACH trial showed good efficacy in steroid-resistant GVHD, both acute and chronic, in adolescents and adults [38,39]. Starting from these results, we could hypothesize that the biological efficacy of ruxolitinib on GVHD may extend to pediatric patients, as shown by some studies, albeit retrospective or limited to case series with variable results. The first concern with ruxolitinib is its efficacy assessment in acute (aGVHD) and chronic GVHD (cGVHD), according to GVHD grade and the organs involved. In one of the largest studies in children, by Uygun et al. [40], results with ruxolitinib treatment were positive in comparison to the best available therapy with an overall response rate of 85% in grade 2–4 acute GVHD patients, with 69% complete response (CR) and 15% partial response (PR). No differences were shown in skin and gastrointestinal responses, allaying concerns about malabsorption of the drug in gastrointestinal GVHD. Further proof of efficacy is the fact these patients were heavily treated for GVHD before ruxolitinib initiation (three or more lines of therapy) and the further fact that corticosteroid use was tapered or discontinued in almost all patients. Similarly, in cGVHD, which is historically associated with steroid-dependence or refractoriness, PR varies between 80 and 89%, with, however, only 8% of patients achieving a complete response [40]. Although this may be a limitation in JAK inhibitor use, it must be taken into account that severe cGVHD, particularly with lung involvement, often had a poor prognosis, with an ineluctable decline in lung function until respiratory insufficiency [41]. Ruxolitinib, in small case series, shows a partial improvement or stabilization of lung function, with an overall reduction of steroid and other immunosuppressor use and better overall survival [42]. Toxicity profiles and doses of ruxolitinib in children vary between different studies, with mostly a low dose initiation (2.5 mg × 2/day in small children and 5 mg × 2/day for those weighing 15–25 kg) and then an escalation until a maximum dose of 10 mg × 2/day if tolerated. The main concerns about ruxolitinib use are cytopenia, infections, and liver toxicity, and the drivability of the drug to reach its therapeutic effect without severe adverse effects remains critical. Furthermore, no studies of pharmacokinetics in pediatric patients are available at the moment. However, studies that initiate therapy at a greater dose for the weight (setting a limit of 15 kg for 5 mg × 2/day dose) have shown better results, especially in aGVHD, suggesting that ruxolitinib’s best effect may be achieved with a greater dose early in the course of disease [40,43,44]. This group of patients also subsequently shows a greater incidence of cytopenia, resolved with tapering of the drug and without clinical consequences. Other adverse effects, besides cytopenia, are liver toxicity and infections. Viral reactivation and fungal and bacterial infections have a slightly higher incidence during ruxolitinib treatment, in particular, cytomegalovirus (CMV) viremia. However, the incidence of severe or life-threatening infection, both viral and bacterial, was found to be low and comparable to other immunosuppressive therapies [40,43]. This differs from a study in adults, where the incidence of fungal infection exceeded other immunosuppressive therapies [38]. Finally, low liver toxicity incidence with no hepatic failure is described in the majority of pediatric studies, although it is shown as a common side effect in adults. Another point of interest is the duration of therapy: studies that assessed response in the first month of therapy showed lower efficacy [45], but when follow-up was expanded, the results were comparable with other studies with a longer follow-up, as shown in Uygun’s study, in which patients’ best responses were stated as occurring at any time after starting treatment with ruxolitinib. This suggests that prolonged therapy may maximize the efficacy of the drug and that early discontinuation of therapy if no response is achieved may be an error. Furthermore, we know from the setting of adult myelofibrosis that early and fast tapering of ruxolitinib could trigger a severe relapse of the disease [46]. This limited evidence may suggest that ruxolitinib should be administered at a dose of 10–20 mg/day, until tolerated, with a successive, slow careful tapering and low dose maintenance in the absence of deterioration or side effects in unresponsive cases [47]. In particular, in cGVHD, although complete response is rare, a slow improvement with a low dose maintenance therapy is suggested, reinforcing the idea that ruxolitinib in cGVHD treatment should be administered for a long period of time to maximize its therapeutic effect and protect patients from irreversible fibrotic damage (Figure 3).

## 4. Infectious Complications

The JAK–STAT pathway has a crucial role in the development and function of the immune system. Moreover, it is well known that ruxolitinib may exert significant immunosuppressive activity through various mechanisms. Loss-of-function (LOF) mutations in JAKs or STATs are associated with immune deficiencies and, as a result, with an increase in susceptibility to infections. LOF in signaling components downstream of γc-dependent cytokines, namely, JAK3 and STAT5B, manifests itself in the most severe phenotype as severe combined immune deficiencies (SCID) [48,49,50]. LOF mutations in STAT1 and TYK2 increase susceptibility to bacterial and viral infections [51,52], while LOF in STAT2 increases the incidence of viral infections and LOF in STAT4 increases the incidence of fungal infections [53,54,55,56]. Due to interference with the JAK–STAT pathway, JAK inhibitors affect several innate and adaptive components of the immune system, such as dendritic, natural killer, T helper, and regulatory T cells, thus carrying out significant immunosuppressive activity [57,58]. Ruxolitinib impairs dendritic cell function, leading to modified CD4+ and CD8+ T cell priming and reduced cytokine production. Inhibition of JAK1 leads to a reduction in IL-12 production, a known T cell stimulating factor. Impairment of dendritic and T cell functions, besides the drop in cytokine production due to ruxolitinib treatment, may increase the rate of viral infections [59,60]. A recent meta-analysis evaluated additional infectious risk in a ruxolitinib-exposed myeloproliferative neoplasm patient population based on phase III randomized control trials and post-marketing surveillance data, including case reports [61]. The authors concluded that there was insufficient evidence to estimate the risk of infection in ruxolitinib-treated patients. This conclusion raises the difficulty of determining the role of ruxolitinib in promoting infection given the fact that myeloproliferative neoplasms are characterized by high rates of infections in themselves, as suggested by Swedish investigations performed before the introduction of JAK inhibitors [62].

One retrospective study examining a cohort of 507 myelofibrosis patients reported that rates of infection were statistically significantly higher in the ruxolitinib-treated cohort than in patients without ruxolitinib exposure (44% vs. 20%, *p* < 0.001). Twenty-two percent of patients experienced 160 infection-related events; 45% of these events were graded as severe, most being bacterial and affecting mainly the respiratory tract [63]. In another retrospective study, including 446 myelofibrosis patients, an incidence rate of 17 cases per 100 patients/year was reported. Respiratory tract infections were also more frequently observed (50%) in this analysis, and bacteria were the most frequent etiological agents (68.9%). Interestingly, the rate of infections tended to significantly decrease over time: 14% of patients developed the first infection within 6 months, 5% between 6 and 12 months, 3.7% between 12 and 18 months, and 3.4% between 18 and 24 months (*p* < 0.0001) [64]. Unlike the data reported in the first two articles, another recent dual-center study reported no difference in risk of infection between ruxolitinib-treated and naïve patients with myelofibrosis (*p* = 0.466) and myeloproliferative neoplasms (*p* = 0.152) [65].

The increase of ruxolitinib-associated mycobacterial tuberculosis was investigated in a retrospective pharmacovigilance review based on the FDA Adverse Events Reporting System, reporting a significant odds ratio (OR) of 9.2 for developing the typical mycobacterial tuberculosis and an OR of 8.3 for atypical mycobacterial infections [66]. Therefore, the European Conference on Infections in Leukemia (ECIL) guidelines recommended always performing a screening with a tuberculin skin test or, preferably, an IFN-γ release assay before starting ruxolitinib if there is a tuberculosis history and significant risk factors are present. Furthermore, during ruxolitinib treatment, a regular follow-up, aimed at early diagnosis of tuberculosis, is also advisable, followed by an appropriate therapy when required [67].

Infectious complications, reported in HSCT recipients treated with ruxolitinib for steroid-refractory GVHD, are mostly viral replication, usually due to CMV, Epstein–Barr virus, adenovirus, and BK virus [40,68,69,70]. Bacterial infections were found to be the next most common infectious event, with a reported bacteremia rate of 42% [71,72]. The increased infection risk among acute steroid-resistant GVHD patients may be due to factors induced by the underlying pathology. Patients receiving ruxolitinib are already heavily pre-treated with high-dose steroids and second- or third-line immunosuppressive drugs. The common feature of these patients is that they have an extremely deficient immune system due either to treatments received or to post-transplant poor graft function.

## 5. Clinical Experience of Ruxolitinib Treatment

Twelve pediatric HSCT recipients from IRCCS Burlo Garofolo of median age 5.8 years (range, 1.1–17.8 years) have been treated with low-dose ruxolitinib in the past two years. Four patients (25%) were under two years old (median age 19 months, median weight 10.7 kg). Out of 12 patients, 11 had GVHD: 5 patients had cGVHD and 6 aGVHD. All but one chronic and two acute GVHD cases were related to donor lymphocyte infusion (DLI). One patient was treated for early-onset idiopathic pneumonia syndrome (IPS).

Out of five patients with extensive cGVHD, three had a scleroderma-like diffuse skin disease and two gathered a multisystemic appearance with severe lung involvement. Two patients treated for the acute form had gastrointestinal involvement grade 4, and four patients had skin and liver grade 2 GVHD. All patients treated with ruxolitinib were suffering from steroid-refractory or steroid-dependent disease. In all cases, the decision to start ruxolitinib treatment was interferon (IFN) signature-guided. The mean IFN signature score was 8.3 (normal range < 2.0), with IPS as a unique outlier (IFN signature score 34.2). 

Two patients with chronic pulmonary GVHD and two patients with aGVHD grade 4 started ruxolitinib in the third or following line, after steroid, mycophenolate mofetil, infliximab, rituximab, basiliximab, extracorporeal photopheresis, fludarabine, and antithymocyte globulin treatments in various combinations. The median delay of ruxolitinib therapy was 37 days (range 31–43 days) after evidence of steroid-refractoriness. The other eight patients started ruxolitinib in the first line after steroids at a median of 7 days (range 3–12 days) from GVHD onset.

All patients started ruxolitinib treatment at 2 doses/day. The initial dose for patients with extensive cGVHD and aGVHD grade 4 was 10 mg twice daily (all patients had a bodyweight > 25 kg). After the clinical symptoms improved for at least one week, ruxolitinib was tapered to 5 mg twice daily. Further dose reduction to 2.5 mg × 2/day was performed in case of complete response for aGVHD, defined as full resolution of all symptoms, or partial response for cGVHD, which required symptom relief (a score for organ improvement of at least 1) [73].

Four patients with aGVHD grade 2 received an unmodified dose of 2.5 mg × 2/day regardless of body weight. A 20-month-old toddler suffering from IPS with a bodyweight of 11 kg received 2.5 × 3/day of ruxolitinib.

All 12 patients had a response to ruxolitinib treatment. Five patients (41.7%) who received an unmodified dose of ruxolitinib achieved complete remission (CR) after an average of 3 weeks. The median duration of treatment was 122 days (range 59–188 days). Four patients maintained CR after immunosuppression discontinuation (minimum follow-up was two months). One patient treated for aGVHD grade 2 relapsed 11 weeks after ruxolitinib discontinuation. Two patients were treated for aGVHD grade 4 for 19 and 21 weeks. They were still in CR after 11 and 5 weeks, respectively, after immunosuppression interruption. All patients treated for extensive cGVHD, including those with pulmonary involvement, are still in ruxolitinib maintenance, 2.5 mg × 2/day, with stable disease after significant initial improvement.

Transitory dose-dependent hematological toxicity and dyslipidemia were observed only in two patients (16.7%) who received the ruxolitinib loading doses of 10 mg × 2/day. None of the patients treated with low doses of ruxolitinib experienced hematological adverse events. The overall rate of CMV reactivation was relatively high: nine patients (75%) had at least one episode of CMV antigenemia. No CMV reactivations, however, were associated with any clinical manifestations. The patients’ clinical characteristics and ruxolitinib treatment outcomes are summarized in Table 1.

## 6. Repositioning Applications of Ruxolitinib: From Autoinflammatory Disease to Viral Infections

Drug repositioning is important in pandemic conditions. In this context, kinase inhibitors, already authorized for different therapeutic indications, are being studied in clinical trials for viral infections [75]. The use of kinase inhibitors offers many advantages over conventional antiviral drug use, as they can target different viral genotypes or serotypes [75,76], and, in light of the availability of new selective inhibitors, are expected to show fewer side effects.

Among the kinase inhibitors group, JAK inhibitors have shown antiviral action, targeting cellular enzymes exploited by viruses for cell entry [77,78] and relieving the inflammation induced by viral infections, such as COVID-19, characterized by cytokine storm [79].

Ruxolitinib, as a selective inhibitor of JAK1 and JAK2, is able to counteract hyperinflammation and is currently being investigated for use in treating COVID-19 infected patients. The limiting effect of ruxolitinib on cytokine production, indeed, has shown a powerful and rapid relapse in limiting the excessive immune response of severe cases of COVID-19 pneumonia, in which there is loss of pulmonary activity and desaturation (low levels of oxygen in the blood) [80,81]. In general, the use of ruxolitinib has been authorized in COVID-19 patients with respiratory insufficiency, and clinical evidence has suggested that this inhibitor of JAK1 and JAK2 can reduce inflammatory pulmonary reaction and potentially prevent the use of intensive care. Several studies have demonstrated that the use of ruxolitinib reduced the time of recovery from lymphopenia [82,83] and led to a decrease in COVID-19 inflammation score (CIS) [84] and in IL-6 [85] and CRP levels [86]. Furthermore, the clinical improvement of respiratory symptoms in patients treated with this oral tyrosine kinase inhibitor was shorter than in the control groups and without progression from non-invasive ventilation to invasive assisted ventilation [82,85,87]. The use of ruxolitinib was associated with a moderate safety profile and the more apparent side effect was the onset of moderate to mild anemia [82,84]. Other side effects, such as increases in alanine amino transferase, were identified in a smaller number of patients, but it was difficult to determine whether ruxolitinib was the real cause because of the concomitant use of other treatments and the effects of COVID-19 infection itself.

Currently, a total of 19 studies have been enlisted under clinicaltrials.gov (https://clinical-trials.gov/ct2/results?cond=ruxolitinib+and+covid&Search=Clear&age_v=&gndr=&type=&rslt= (accessed on 9 March 2022) that involve the use of ruxolitinib for treating COVID-19 either as a single regimen or as part of a combined regimen with other drugs. Out of these 19 studies, 3 studies have not started recruiting subjects, while 3 studies are already recruiting subjects; the subjects for 14 studies are available. Amongst these, five are completed and two have been withdrawn [88].

Furthermore, it has been demonstrated that ruxolitinib also has an antiviral action against HIV and Epstein–Barr viruses [89,90,91,92,93,94]. In vitro experiments have shown that ruxolitinib is able to block viral replication in lymphocytes and macrophages, perhaps reducing the phosphorylation of STAT proteins, and inhibit the reactivation of latent HIV-1 [90,91]. It has also been demonstrated that this drug can reduce astrogliosis in the brains of mice with encephalitis [92] and decrease the production of pro-inflammatory cytokines caused by Ingenol derivatives, without influencing the latency reversal induced by these treatments [88].

In the context of viral diseases, some studies have also suggested the use of this inhibitor to counteract chronic active Epstein–Barr virus infection. Ruxolitinib showed the capability to suppress inflammatory cytokine production and STAT-3 phosphorylation in an in vitro study [93] as well as the ability to reduce the copying of EBV DNA and the size of the spleen of a 9-year-old patient affected by chronic active Epstein–Barr virus [94].

## 7. Conclusions

Clinical evidence in the pediatric population has demonstrated the significant preventive anti-inflammatory capacity of ruxolitinib when administered at a minimum dosage (2.5 mg twice daily); at the molecular level, this condition corresponds to a partial blockage of JAK receptors, which limits the hyper-inflammation associated with STAT phosphorylation (Figure 3). The anti-inflammatory role played by ruxolitinib has allowed the repositioning of this drug in other areas clinically characterized by cytokine storm, e.g., severe cases of COVID-19 [40,41]. Finding modulators of target cytokines specific for cGVHD that involve inflammatory activation has been very important, as it has allowed the application of precision medicine in this field. Since recent studies have shown crosstalk between different pathways involved in the activation of the inflammatory process, it appears crucial to investigate the activity of molecules stimulated or inhibited by different drugs and to identify common mechanisms underlying the pathogenesis of GVHD. 

For the treatment of GVHD, ruxolitinib has shown good clinical efficacy in adult patients and could also be applied to pediatric patients, judging by the series of available cases and our clinical experience. However, the toxicity of the drug is not negligible and evaluations of the indicated pro-kilo dose, duration of therapy, and reduction are still needed. Considering the growing claims in the pediatric environment for HSCT, clinical efficacy studies and pharmacokinetic and pharmacodynamic studies will help to achieve greater manageability of the drug, lower transplant-related mortality, and general greater safety of the HSCT procedure.

## Figures and Tables

**Table 1:** Patients and GVHD characteristics and ruxolitinib outcomes.

Variables	Value
Age at treatment (median, years)	5.8 (1.1–17.8)
Acute GVHD (number, %):	6 (50.0)
Grade 2	4 (33.3)
Grade 4	2 (16.7)
Severe chronic GVHD (number, %):	5 (41.7)
Lung involvement	2 (16.7)
Idiopathic pneumonia syndrome (number, %)	1 (8.3)
Treatment before ruxolitinib ^a^ (number, %):	
1	8 (66.7)
2	0
≥3	4 (33.3)
Response to ruxolitinib ^b^ (number, %):	12 (100)
Complete response:	7 (58.3)
Acute GVHD all grade	6 (100)
Idiopathic pneumonia syndrome	1 (100)
Partial response:	5 (41.7)
Chronic GVHD	5 (100)
Ruxolitinib-related adverse events (number, %):	9 (75.0)
Hematological toxicity: [74]	2 (16.7)
Grade 2–3	2 (16.7)
Grade > 3	0
CMV reactivation	9 (75.0)
Dyslipidemia	2 (16.7)
Liver toxicity	0

^a^ Other than the drugs used in GVHD prophylaxis. ^b^ One month for aGVHD and three months for cGVHD.

## Data Availability

Data sharing not applicable.
